# VLBI observations of GNSS-satellites: from scheduling to analysis

**DOI:** 10.1007/s00190-016-0992-8

**Published:** 2017-01-16

**Authors:** Lucia Plank, Andreas Hellerschmied, Jamie McCallum, Johannes Böhm, Jim Lovell

**Affiliations:** 1 University of Tasmania, Private Bag 37, Hobart, 7001 Australia; 2 Technische Universität Wien, Vienna, Austria

**Keywords:** Space tie, Co-location in space, Very long baseline interferometry (VLBI), VLBI satellite tracking, Global navigation satellite systems (GNSS)

## Abstract

The possibility of observing satellites with the very long baseline interferometry (VLBI) technique has been discussed for several years in the geodetic community, with observations of either existing satellites of the global navigation satellite systems or of satellites dedicated to realise a space tie. Such observations were carried out using the Australian telescopes in Hobart and Ceduna which, for the first time, integrated all the necessary steps: planning the observations (automated scheduling), correlation of the data and the generation of a series of time delay observables suitable for a subsequent geodetic analysis. We report on the development of new and the adaptation of existing routines for observing and data processing, focusing on technology development. The aim was to use methods that are routinely used in geodetic VLBI. A series of test experiments of up to six hours duration was performed, allowing to improve the observations from session to session and revealing new problems still to be solved. The newly developed procedures and programs now enable more observations. Further development assumed, this bears the prospect of being directly applied to the observation of dedicated space-tie satellites.

## Introduction

VLBI is one of four space geodetic techniques contributing to the International Terrestrial Reference Frame (ITRF), a key product of geodesy. As a combined product, the ITRF heavily relies on ties between the various techniques, which are traditionally determined via classical surveying at co-location sites. These tie vectors differ from space geodesy solutions by up to a few centimetres (Altamimi et al. 2011; Seitz et al. 2012). As an alternative approach, the Global Geodetic Observing System (GGOS) concept—targeting accuracies at the 1-mm level on global scales—includes the possibility of VLBI observations of satellites (Rothacher et al. 2009): observing satellites of the GNSS would directly link the two techniques; another possibility is a dedicated low-Earth-orbiting space-tie satellite, which can be tracked by all techniques: while GNSS, satellite laser ranging (SLR) and Doppler Orbitography and Radiopositioning Integrated by Satellite (DORIS) are well-established techniques, VLBI observations of geodetic satellites are not performed routinely yet.

### VLBI satellite tracking: previous work

The principle of observing space craft with VLBI is not new. It is an established and regularly applied technique for the navigation of spacecraft and a multitude of applications ranging from planetary science to fundamental physics (e.g. Lanyi et al. 2007; Lebreton et al. 2005; Duev et al. 2012; Hanada et al. 2008). These present applications are mostly situated in the fields of spacecraft navigation or astronomy and usually employ quite different observing and processing techniques than those in geodetic VLBI.

Within the geodetic VLBI community, which is widely represented by the International VLBI Service for Geodesy and Astrometry (IVS; Nothnagel et al. 2016), the idea of observing GPS satellites was first discussed as a possibility for phase centre mapping (Corey 2001; Schmid and Rothacher 2003). Proposed missions such as the Geodetic Reference Antenna in Space (GRASP; Nerem and Draper 2011), which have a VLBI transmitter on board a satellite, finally triggered serious tests and research on this topic. Several studies have been performed on general conditions concerning satellite orbits and suitable antenna networks (Plank et al. 2014; Plank 2014), as well as on initial scheduling strategies for observations of GNSS-satellites (Plank et al. 2016). Depending on the satellite orbit and tracking network, Plank et al. (2014) find that when assuming a measurement noise of 30 ps, station positions at the level of a few millimetres in weekly solutions are feasible.

On the observational side, a series of test experiments (Tornatore et al. 2014; Haas et al. 2014; Hellerschmied et al. 2014) have been performed, mostly including antennas in Onsala (Sweden), Medicina (Italy) and Wettzell (Germany). Given the fact that GNSS signals transmit in the L-band frequency range, such experiments had to be performed on telescopes with a suitable L-band receiver, rather than the dual-frequency S/X-band receiver that is commonly used in geodetic VLBI. An exception to this restriction are the antennas in Wettzell, where special equipment allows for sufficient reception of the L1 signal after passing the standard S-band signal path (Kodet et al. 2014). These experiments have prompted new developments in the scheduling and observation strategy (Hellerschmied et al. 2015): today, an experiment of a few hours can easily be planned and is observed on suitable antennas mostly using the standard antenna control systems. Haas et al. (2014) report on the successful correlation of one of these experiments and present phase delays with scatter below 10 ps for intervals of 2 s or larger. Here again the problem is that the standard processing chains used in geodetic VLBI are not prepared for such observations. Despite ongoing development in this direction, final results in terms of a time series of such observations or a geodetic analysis have not been shown so far, to our knowledge.

For the realisation of the frame-tie itself, various strategies are feasible: the two mostly discussed at the moment are the option of orbit determination with VLBI or the determination of station coordinates in a satellite frame and a subsequent comparison with a VLBI frame (e.g. Plank et al. 2016). For the first option, a proper orbit estimation tool will have to be implemented into the analysis software. While the final strategy will depend on the satellite constellation and a matching telescope network, the major issue for current satellite mission proposals is the lack of knowledge about the optimal signal structure and how this can be processed with default station equipment.

The urgency of more research on VLBI observations of satellites is proven by the fact that since September 2015 the APOD satellite mission (Tang et al. 2016), a set of four-cube satellites with one of them equipped with an S/X-VLBI transmitter, is in orbit and active.

### Aim of this work

In this work we present successful VLBI observations of GNSS-satellites, including all tasks from scheduling to analysis. Our aim was to streamline the whole process (Table 1), using as many available procedures and well-established programmes as possible. Technical development and information about the interaction between the unusual signal and the default hardware were further targets of these investigations. For a few test experiments performed on the Australian baseline Hobart–Ceduna an automated scheduling tool was used for the first time to combine satellite targets and observations of quasars for calibration (Sect. 3). We report on our experience on using various standard VLBI hardware and backends in Sect. 4. Using a new a priori delay model, procedures have been developed to correlate the data using the DiFX software correlator (Deller et al. 2007, 2011) and perform the subsequent fringe fitting which is described in Sect. 5. The Vienna VLBI Software (VieVS; Böhm et al. 2012) is used to analyse the observations in a classical way common in geodetic VLBI (Sect. 6). In total, the presented work shall, for the first time, present results from a series of VLBI observations of GNSS-satellites; and, more importantly, the developed process chain now allows for more observations to be performed without complicated preparation and enables data processing including correlation and analysis. This is a first important step towards the final aim of realising actual frame ties.

**Table 1:** The observations begin with the scheduling, which provides two different output files

1. Scheduling	VieVS	
	Input:	TLE
	Output:	VEX, VSO
2. Observation	Ceduna	
	Receiver:	1.2–1.7 GHz
	Diameter:	30 m
	Sampler:	DBBC
	Recorder:	Mark5C
	Hobart26:	
	Receiver:	1.2–1.7 GHz
	Diameter:	26m
	Sampler:	Mark4/DBBC
	Recorder:	Mark5A/Mark5B+
3. Correlation	DiFX, fourfit (AIPS)	
4. Analysis	VieVS	
	Input:	VSO, SP3

The observations themselves are done with the Ceduna and Hobart telescopes equipped with L-band receivers and standard geodetic samplers, formatters and recorders. The correlation is done using the DiFX software, and the total delays are generated in fourfit. VieVS is used to perform the final analysis

## Overview of experiments

**Fig. 1:**
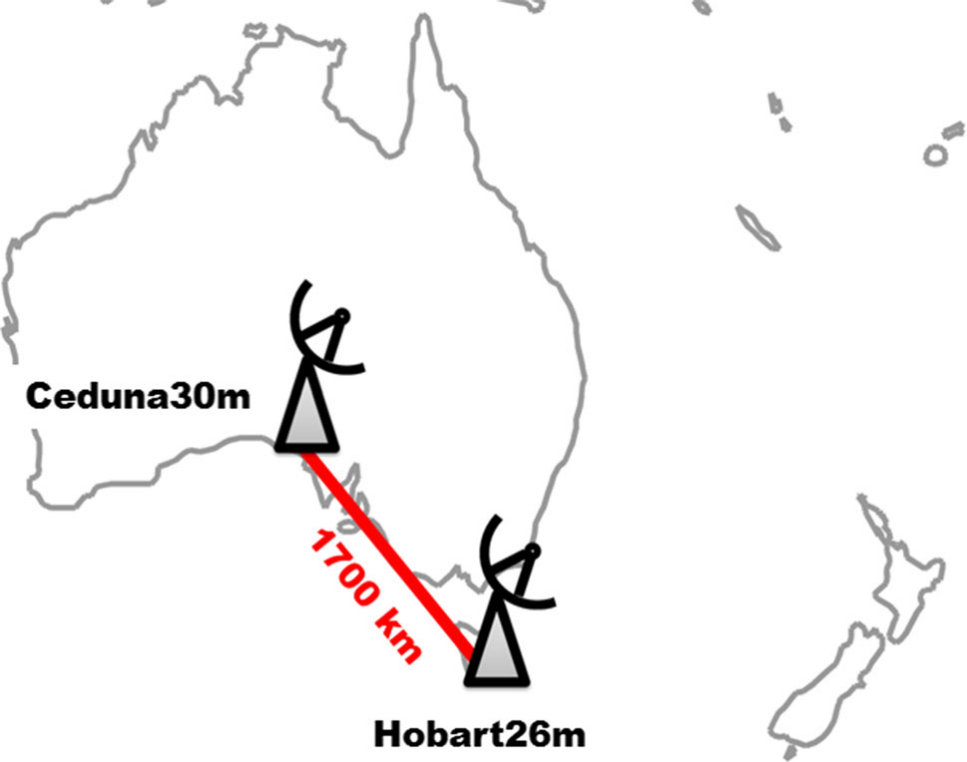
Observations performed with antennas in Hobart and Ceduna, a baseline of about 1700  km

The experiments described in this paper were done using antennas owned by the University of Tasmania, the 26-m radio telescope in Hobart, Tasmania, and the 30-m antenna in Ceduna, South Australia (McCulloch et al. 2005). The baseline is about 1700 Km long (Fig. 1). Both antennas are equipped with L-band receivers and state-of-the-art antenna control and recording backends (see Sect. 4 for details). First tracking tests were done in June 2015, with the aim to confirm the ability of the telescopes to reliably track the satellites and to ensure that the L-band receivers were not saturated by the GNSS transmitters. The first VLBI experiment was done on 28 June 2015, being referred to as experiment 179a hereafter (Table 2). After successful correlation of this first block of 4-h observations, two more sessions (236a, 238a) were made in August 2015, with modifications in the recorded frequencies. Results of 236a are described in Hellerschmied et al. (2016). A final set of experiments was done in May 2016 (126b, 131a, 132a), generating the data for most of the results presented in this paper.

**Table 2:** List of VLBI satellite tracking experiments on the Hobart–Ceduna baseline

Experiment code	Date	Time	Targets		Comments
		(UT)	GPS	GLONASS	
–	June 15	–	$\checkmark$	$\checkmark$	Tracking tests
179a	28.6.15	18–20	$\checkmark$	$\checkmark$	16 satellites,
					Change frequency for each satellite
236a	24.8.15	12–16	$\checkmark$		Fixed frequencies, dual polarisation
238a	26.8.15	12–16	$\checkmark$	$\checkmark$	Fixed frequencies, dual polarisation
126b	5.5.16	17–23	$\checkmark$		DBBC in Ho; no Mark4 data
131a	10.5.16	17–23	$\checkmark$		Redundant recording (DBBC + Mark4) in Ho
132a	11.5.16	17–23	$\checkmark$		Not observed due to high winds

In Table 1 we illustrate the flow chart of one observation, with each task being described in detail in the following sections.

## Scheduling

The scheduling depicts the planning of the observations, in determining which antennas shall observe which satellite at what time. In geodetic VLBI, this is usually done with dedicated software (e.g. SKED or VieVS; Gipson 2016; Sun 2013), balancing a variety of preferred scheduling options. Key features of a scheduling tool are the proper calculations of source visibilities, accounting for the individual capabilities of antenna sensitivity, slewing rates and source strengths. The result are schedules in a standard format from which each participating station extracts its relevant information. For stations using the NASA Field System,^1^ this step is performed by *drudg*. The station-specific schedule file contains all the commands necessary to carry out the observation including the antenna steering, signal chains and sampler configuration, together with the recording mode which is also defined when scheduling.

For this work, we used the satellite scheduling module of VieVS. As described in detail by Hellerschmied et al. (2015), the observation configuration to near-field targets has been successfully implemented and tested. All antenna specifications and steering are treated as for the standard geodetic scheduling (Sun 2013; Sun et al. 2014) while coordinates for the satellite targets are implemented via public two-line element (TLE) orbit data sets. Due to the high angular velocity of satellites, compared to the sidereal pace of astronomical sources, the scheduling becomes much more complex: the observation timing is much more critical, and additional criteria have to be taken into account, such as the limited antenna slew rates during data acquisition, the time required for calibration or for the recorders to either start, stop, or check the recorded data. The supported output formats are station-specific VEX-files (VLBI experiment format) for observation (Sect. 4), a combined VEX-file for correlation (Sect. 5) and a VSO-file for analysis (Sect. 6).

### Scheduling development

In the course of this work, a variety of settings were applied and tested: for the first experiment, 179a, 16 satellites of both the GPS and the GLONASS were observed. The recorded frequencies were chosen individually for each track, as the transmitted frequencies of the GLONASS satellites are different for each satellite. The frequent changes to the backend frequency selection were identified as causing significant and variable delay offsets per satellite. The backends, in particular the analogue Mark4-rack in Hobart, show frequency-dependent instrumental delays. Without a proper calibration across the whole frequency band, this instrumental delay cannot be separated from the geodetic signal. Consequently, all further sessions were done with a fixed frequency setting, centred on the transmitting frequencies in the L1- and L2-bands, which in the case of GPS are 1575 MHz (L1) and 1227 MHz (L2).

Besides the satellite signal, we also observed strong quasars for use in estimation of the clock model and the instrumental delays. In order to increase the bandwidth for the quasar scans, two additional bands were added. For the final set of experiments (126b, 131a, 132a), quasar blocks were added about once per hour, observing four to five quasars per block.

Given the strength of the GNSS signals, clear cross-correlation detections could be made with sub-second integration times. As such, the scan durations for GNSS sources were chosen to allow for a reasonable number to be observed in a scheduling block of typically 50 min. For the quasars, a fixed one minute integration time was chosen rather than trying to optimise for a specific signal-to-noise ratio (SNR) threshold. The satellite positions were provided to the telescope via topocentric right ascension (RA) and declination (Dec), and a suitable update interval for this stepwise tracking had to be defined. Various tracking intervals were trialed, and 10 s was found to be suitable for observing GNSS-satellites with these telescopes.

After the analysis of the first session 179a, it turned out that re-observing satellites periodically is useful in assessing the quality of the a priori delay model, which in turn helps to interpret the residual delays in analysis. Hence, from 236a/238a on, a set of about five satellites was chosen for each session which were observed several times.

### Automated scheduling

All initial sessions in 2015 (up to 238a) were done using the interactive scheduling mode, actively selecting each individual satellite scan (Hellerschmied et al. 2015) when preparing the schedule. Having gained the necessary experience to determine the scheduling requirements, in 2016 this process was automated: VieVS now allows for automated scheduling of combined observations of satellites and quasars. This is important, as a manual selection of scans is not practicable for experiments of several hours or more.

The new automated scheduling mode implements a so-called station-based scheduling approach (as described e.g. by Sun et al. 2014), optimising the sky coverage at individual stations equally for quasars and near-field targets. While the interactive mode is good for shorter test sessions as it provides full control on the source selection, the automated mode determines the best scan configuration —in terms of a good sky coverage and a minimum time spent on slewing—without manual interaction. This mode is able to schedule alternating blocks of quasar and satellite scans in a defined time ratio.

The schedule is created automatically after being provided with a list of satellite targets and suitable quasars, together with the desired scan durations for the satellites and a ratio for the time allocated for the satellite and quasar sources. Further scheduling parameters, such as the minimum time interval for re-observing a source and the time window for the sky coverage optimisation, can also be provided and adjusted.

**Fig. 2:**
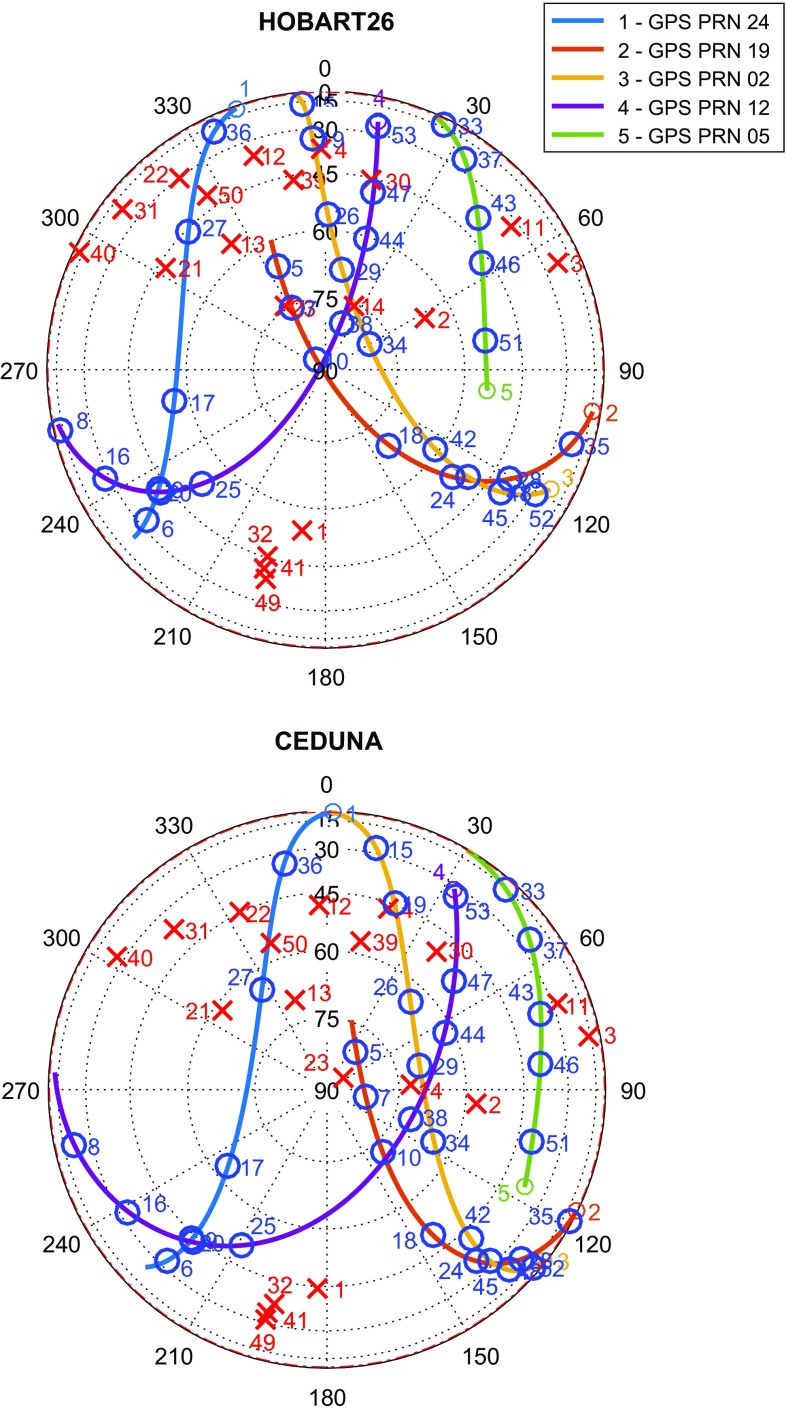
Skyplots for the participating stations Hobart26 (*top*) and Ceduna (*bottom*) of the 126b experiment (May 5, 2016, 17:00–23:00 UTC). *Blue* circles and *red* crosses tag satellite scans and quasar scans, respectively, with the scan number next to it (53 scans in total)

The scheduling strategy in the last set of experiments in 2016 (126b, 131a, 132a) aimed to observe a limited number of GPS satellites (usually five) several times in each session. The re-observations help to reveal systematic behaviours in the data acquisition and to interpret the delay results. Furthermore, selected quasars—used as calibrators—were observed periodically by scheduling a 10-min block of quasar scans every 50 min. The resulting observation plan for the 126b session is illustrated in Fig. 2.

As the 131a and 132a sessions were planned for the same time on two consecutive days, it was easily possible to reproduce the 131a schedule for the next day, observing the same targets in exactly the same order. This constellation provides interesting possibilities to study the stability of our process chain. Unfortunately, 132a was not observed due to high winds at Hobart.

Since the used scheduling tool is fully embedded in VieVS, it can be directly connected to a simulation tool allowing for studies about the optimal schedule and expected accuracies on final products (e.g. the frame tie). Plank et al. (2014) and Plank et al. (2016) present examples of such studies.

## Observation

### Technical specifications

The nominal operating range of the used L-band receivers is between 1.2 and 1.7 GHz, measuring in orthogonal linearised polarisations. The antenna sensitivity—represented as the system equivalent flux densities (SEFD) measured in Jansky (Jy)—is $\sim$400 Jy for Hobart and between 1200 and 1600 Jy for Ceduna, over the observed frequency range. The Ceduna telescope is an azimuth/elevation (AzEl) mount with a slew speed of $40^{\circ }$/min in both axes. Hobart is an XY-mount telescope with maximum slewing rates of $40^{\circ }$/min in each of the X and Y drives. Both antennas have extremely slow acceleration, which, in the present case of a 10-s repositioning interval, leads to a largely continuous motion during the tracking.

Hobart is more suitable for tracking satellites, because XY-mount antennas do not have a so-called key hole in zenith direction contrary to AzEl antennas such as Ceduna. Therefore, they have complete tracking capability through the zenith. The keyholes of XY-mount type antennas are in the horizontal plane, which does not affect satellite tracking beyond creating a horizon mask.

### Frequency set-up

As mentioned previously, the frequency set-up was changed for the various experiments. Throughout all the experiments, the recorded data consisted of eight intermediate frequency (IF) channels of 16 MHz bandwidth using 2-bit sampling.

Studying the results of the first sessions, we found different offsets in the various bands. In order to distinguish between instrumental, satellite-specific or modelling effects, we decided to have fixed sky frequencies defined in a common observation mode for all targets for the next experiments. This was also motivated by the experience that the Mark4 rack at Hobart did not operate reliably when many frequency changes were made. In an additional modification, we opted to concentrate on GPS observations only. While the signals of GLONASS observations were also successfully correlated, the results were somehow not as good as for the GPS data (also see Sect. 5.3).

**Fig. 3:**
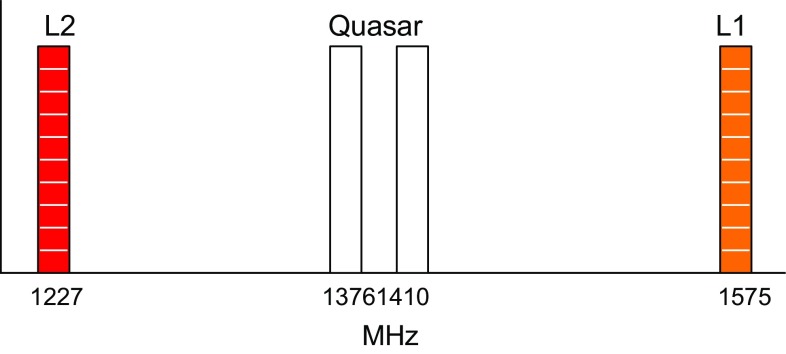
Observed frequencies in 126b and 131a. Each band is 16 MHz wide and was recorded in dual polarisation. One frequency band was each centred on the GPS L1 and L2 transmission. The quasar signal was expected to be present in all four bands

A complication is the fact that while the emitted satellite signal is circularly polarised, the Hobart and Ceduna telescopes have only linearly polarised receivers. With the aim to reconstruct the full signal, we decided to record both polarisations. For the final experiments (126b, 131a), the signal was recorded in eight channels, using four different frequencies (Fig. 3). One frequency is then recorded twice, once in (local) X polarisation and a second time in Y polarisation. The centre (sky) frequencies were set on the GPS L1 (1575 MHz) and L2 (1227 MHz) frequencies, and two additional channels were added in between at 1376 and 1410 MHz, with the aim of estimating multi-band delays for the quasar scans.

### Tracking

For the observation itself, the two individual VEX-files were used at the two stations, Ceduna and Hobart. It is necessary to use station-dependent VEX-files, because the source positions of the same satellite (defined in topocentric RA/Dec) differ between sites. The tracking was done using the NASA Field System by commanding the satellite positions in stop–start mode. New sources were commanded every 10 s, also referred to as stepwise tracking approach. For one satellite scan of typically 5-min duration, the antennas were steered to a nominal position about 1 min before the actual recording was meant to start. Once the satellite comes into the antenna beam, the automatic gain control (AGC) in the DBBC can adjust smoothly to optimal power levels for when the recording starts. The Mark4 rack used fixed attenuator settings instead, which were confirmed as valid during this pre-observation interval. Once the scan started, the recording is continuous throughout the whole scan, with minimal station checks carried out during the observation. After the experiment, the Ceduna data were copied to a portable hard drive and shipped to Hobart for correlation. For the six hour experiment, about 1-TB data were recorded per station. In Hobart, by default the Mark4 rack and a Mark5A recorder were used. For 126b and 131a the data in Hobart were additionally recorded in parallel with a DBBC and Mark5B+, allowing comparisons between redundant data (see Sect. 5).

In general, the stepwise tracking approach with a 10-s re-position interval worked well for the telescopes and the observed GNSS-satellites. As described in the next section, the received signal strength was quite stable over the entire scan. The tracking accuracy was also confirmed by comparing the scheduled antenna position with the actual position the antenna had during the observation. We find good agreement (within 0.1 degrees or a fifth of the beamwidth at worst) for most of the scans. During all scans of 126b and 131a, we found two tracks where the satellite was close to zenith and Ceduna could not keep up with the required slew rate in azimuth. Small improvements can be made by re-estimating the actual slew rates and accelerations of the telescopes, which were late on source on several occasions although these late starts did not cause any significant loss of data.

There were several large gaps in the data in 126b and 131a due to an intermittent problem with the antenna drives in Ceduna and high winds at both sites.

### Signal

**Fig. 4:**
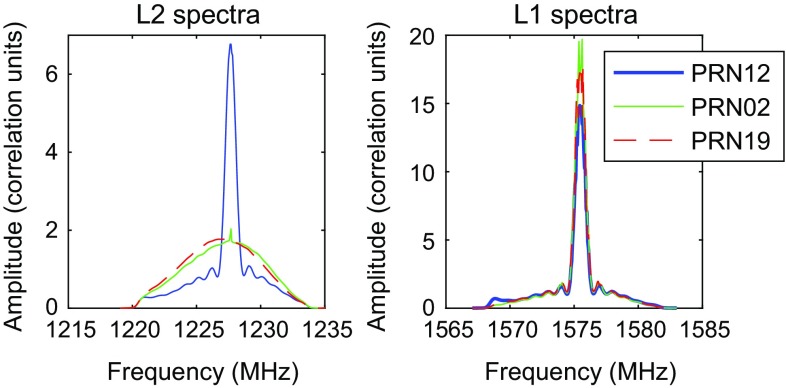
Signal spectra (autocorrelation) of three satellites observed during 126b in L2 (*left*) and L1 (*right*). The amplitudes are normalised using template spectra on the quasar sources

When observing an artificial satellite signal rather than the radio emission of a natural radio source, the signal is vastly stronger and it is possible to actually see the signal during observation without real-time correlation. As it is transmitted on discrete frequencies, it is possible to use a spectrum analyser to observe this directly from the antenna output. This was done in our initial tests to check for signs of saturation in the front end low-noise amplifiers (LNAs), and in subsequent observations we could clearly see the satellites coming in and out of beam when the antenna was slewing from one target satellite to the next, and during the minute-long pre-observation interval. The signal with its main peak and the side lobes was clearly visible. This is illustrated in Fig. 4, with autocorrelation spectra of the recorded data showing the typical appearance of the two main transmission frequencies for three different satellites during 126a.

The L1 spectrum is dominated by a clear peak corresponding to the spread spectrum of the transmissions together with a broader carrier. It is consistent between the different GPS satellites, but there are some noticeable differences in the L2 spectra. In the example shown above, the PRN12 satellite shows a strongly peaked spectrum while the other two show only the broad carrier. The reason for these differences has not been identified yet and is possibly related to actual differences in the transmitted signals of the various satellites.

## Correlation

The data were correlated using the DiFX software correlator (Deller et al. 2011). Initial processing was performed using version 2.4.1, but all results presented here have been produced using the trunk version (v7326), current as of June 2016. DiFX requires a schedule file in VEX format for information of the configuration of the experiment including frequency set-ups, antenna scheduling and source information. In our case we use a combined VEX-file, which is one output from the scheduling module (Sect. 3). This combined file is essentially the merger of the individual station-specific VEX-files that were used to carry out the observations, expanding all blocks ($MODE section, etc.) to include both stations. For $SOURCE section, one telescope (Ceduna) was chosen to act as the template for the source coordinates, and both telescopes were added to the scans in $SCHED section. When the VEX-file was processed through DiFX’s vex2difx and calcif2 processes, input model (IM) files were created treating the GNSS sources using the standard (quasar) model in Calc. Correlation and conversion of the output data to both FITS and Mark4 format were performed after replacing the IM-files with the near-field model (Sect. 5.1). Quasar observations were used for initial fringe fitting to establish a clock model for the correlator.

### Near-field delay model

With the satellite being at finite distance to the antennas, the assumption of planar wave fronts that are usually sufficient in quasar VLBI does not hold any more. In VieVS a proper iterative light time solution of travel times is performed (e.g. Moyer 2003; Plank 2014). The fact that both station coordinates and satellite positions are given in a geocentric system simplifies the procedures. We use satellite orbits in the SP3-file format, as provided by the International GNSS Service (IGS; Dow et al. 2009). In geodetic VLBI, one observation —or delay in signal arrival times between two receiving antennas—usually refers to the time of signal reception at station one. This was adopted for the satellite observations.

For the correlation model, one needs to provide a so-called geocentric model. This is the delay between each station and the geocentre. In VieVS this is simply realised by replacing station one with the geocentre.

The practical realisation uses the VSO-file created in the scheduler and generates the geocentric model in VieVS. This file format is essentially a list of all observations with time epoch, participating stations and the (modelled or observed) time delay. The result is a geocentric delay for each station, at the time epoch of each scan (every 10 s). This model is further converted into a 5th-order polynomial valid over 2 min and finally replacing the erroneous far-field quasar delay in the IM-files.

### Correlation, fringe fitting and total delay

The correlation of the observations was carried out on a small computing cluster running DiFX, based at the Mt Pleasant observatory. A subset of observations was re-correlated on the Vienna Scientific Cluster at Technische Universität Wien, using a 64-bit system rather than a 32-bit system and allowing for a cross-check of the correlation results. The initial correlation was performed with 0.25-s integration time and high spectral resolution (7.8125 kHz) to allow the investigation of high residual delays and rates. The accuracy of the near-field model made this unnecessary, and subsequent re-correlations used coarser spectral resolutions. For the results presented in this paper, bandwidths of 62.5 kHz (256 channels over 16 MHz) have been used uniformly with integration times of 0.1 s. The high temporal resolution has been retained in order to investigate short timescale variability in the signal.

It is worth noting that the GNSS signals are almost overwhelmingly strong in the measured visibilities. Typical correlation amplitudes for the GNSS sources on the Ceduna–Hobart baseline are almost 100 % for the L1-band and above 50 % in the L2-band, compared to a few per cent which might be possible for a strong quasar source.

Initial fringe fitting on both the quasar and GNSS sources was carried out with the AIPS package (Greisen 2002), using the FRING task. We have also adopted the use of fourfit, part of the Haystack Observatory Postprocessing System (HOPS)^2^ and the standard for geodetic VLBI. A major advantage of using fourfit is the provision of total delays in the geodetic sense, namely referenced to reception at the first station at integer second time. All fringe fitting was carried out in single-band mode.

**Fig. 5:**
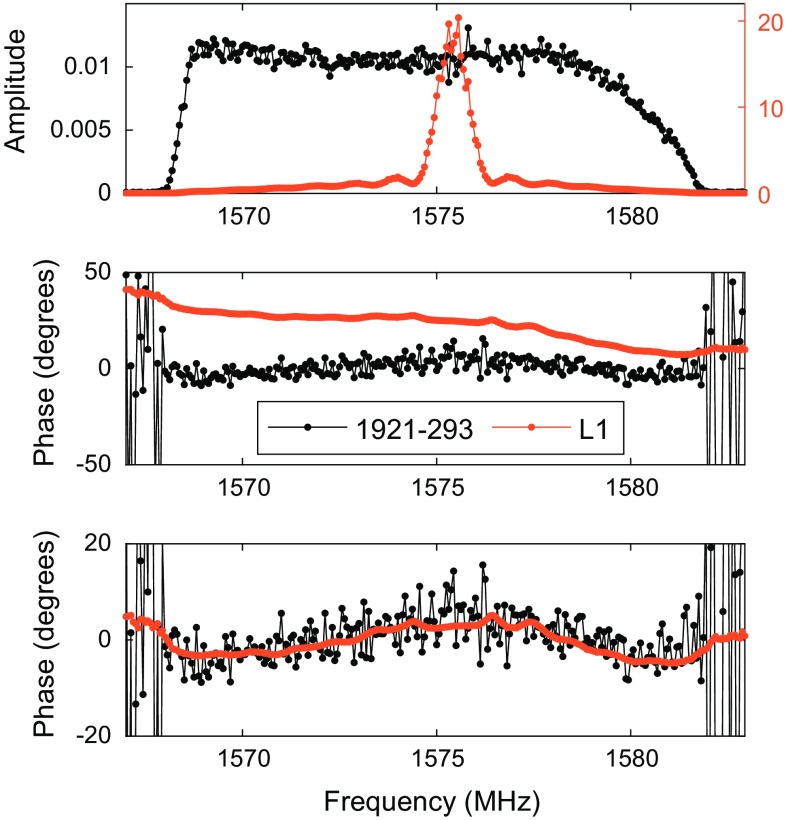
Typical cross-correlation spectrum from 126b in L1, averaged over one scan (10 s for the satellite, 60 s for the quasar). The *top panel* shows amplitudes of the GPS source in orange (scale on the *right*) and the quasar 1921-293 in black (scale on the *left*). The *middle panel* shows raw phases before fringe fitting, and the *lower panel* shows residual phase after delay calibration (per scan). The residual bandpass phase shows some structure, mostly towards the band edges. There is good agreement between the quasar and satellite response

**Fig. 6:**
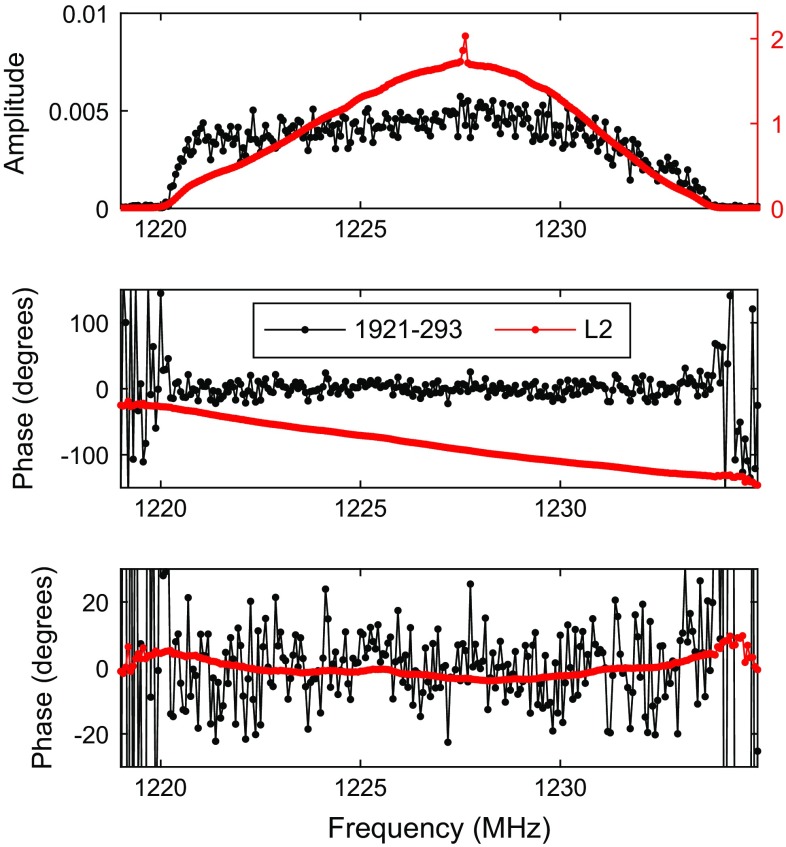
Same as Fig. 5, for L2

Typical cross-correlation spectra are shown in Figs. 5 and 6, for both satellite and quasar data. The extreme ends of the bandpass have essentially no signal and do not contribute strongly towards the delay estimation. The satellite signal shows a continuous phase against frequency over the scan, including the spread spectrum peak in L1. The residual bandpass phase shows some structure, largely towards the band edges. Bandpass calibration has not been performed on these data, as this is not part of the fourfit processing. However, there is good agreement between the quasar and satellite residual phase which suggests that there is no obvious systematic difference due to the signal strengths. The absence of any nonlinearity in the response is encouraging and makes bandpass calibration a viable technique for these observations.

All delays measured through fourfit are derived using the entire 10-s scan on the satellite and are fitted using the entire recorded bandpass of 16 MHz. To investigate rapid variability, we have used AIPS to process the data which has revealed a number of rapid variations in both amplitude and delay. Hereby the delay is a residual delay with respect to the a priori delay model. In order to avoid confusion with the residual delay of Sect. 6, it will be called *fringe delay* hereafter.

**Fig. 7:**
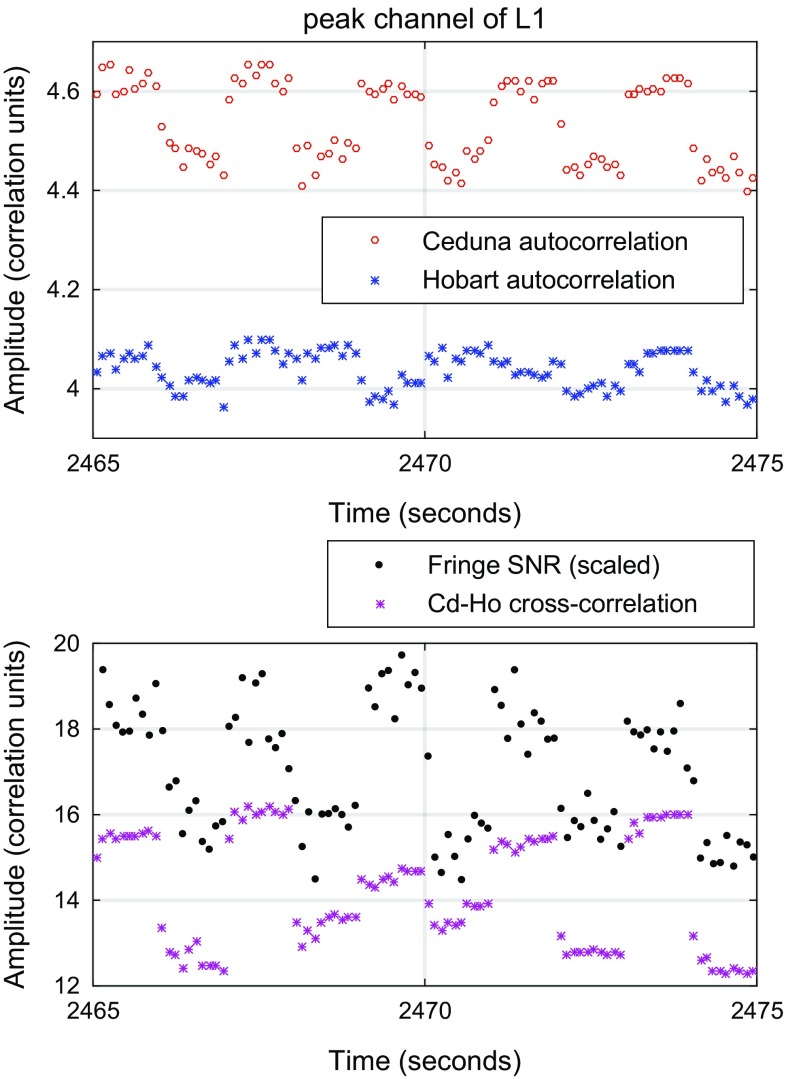
Gain variation in 126b as seen in the auto-(*top*) and cross-(*bottom*) correlation. The amplitudes vary with a period of 2 s, also causing jumps in the fringe SNR at the same periods

A prominent feature of the visibilities is a large amplitude variation with a period of 2 s. This is seen in both the auto- and cross-correlations and is illustrated in Fig. 7. The amplitudes show a clear bifurcation between two levels, with transitions occurring on integer second boundaries. These variations are linked to the AGC systems within the DBBC, which adjust attenuation and sampling thresholds on this timescale. Data recorded using the analogue Mark4 rack at Hobart with fixed attenuators do not show this behaviour.

The gain variations caused by the DBBC’s AGC struggling to maintain an optimal sampling level lead in turn to variations in the estimated single-band delay. The varying gain affects the relative amplitude of the different parts of the spectrum, emphasising or de-emphasising the central band of the transmitter. In the L1-band where the transmitter’s peak is most prominent, the delay ”noise” caused by these variations can reach a peak-to-peak amplitude of almost 1 ns.

**Fig. 8:**
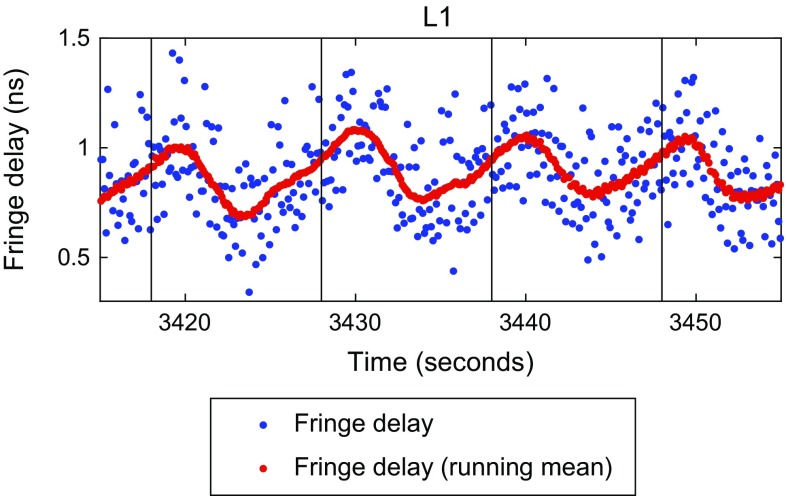
High time resolution of fringe delays in L1 for 126b. Data points are every 0.1 s (*blue*) and a running mean using a 2 s wide boxcar window (*red*). Scan start times are indicated with vertical lines. A variation with the 10-s stepwise tracking interval is clearly seen

At sub-scan integration times, the stepwise tracking used in these observations does create observable variations in both amplitude and measured delays. While the amplitude variations are generally small ($\approx$1%) due to the relatively small angular offsets between steps compared to the beam size, the peak-to-peak variations in the delay are typically between $\approx 40$ and 400 ps. Figure 8 shows an example with some of the largest variations. Using an integration time of the entire scan length of 10 s, we assume that this tracking effects largely cancel in the data used for the subsequent analysis.

There are several possible options to mitigate the spurious delay variations linked to the gain. Disabling the AGC loops prior to the start of the recordings in the DBBC should improve the issue although real variations in the power level will still cause gain errors due to non-optimal sampling. The amplitude of these errors is expected to be comparable with those caused by the stepwise tracking. Fringe fitting to only the peak channels of the spectrum in order to reduce the effect of the varying gain was also tried, but didn’t yield satisfying results. Recording the data with 8 bits instead of 2 would greatly improve the dynamic range of the recorded data, which should reduce the effects of compression from the strong transmitter tones. Given the high-resolution variations with the 10-s tracking interval, continuous tracking of the satellite would be favourable in the future.

After fringe fitting was completed, results were extracted and loaded into MATLAB for analysis in VieVS. The data contained the total delays (a priori model plus residual fringe delays) in each observed band and polarisation.

#### Polarisation issue

The signal transmitted by the satellites is circularly polarised, while both Hobart26 and Ceduna are equipped with receivers with orthogonal linear feeds. Nominally, when we record both polarisations we should be able to reconstruct the full signal. However, this has proved challenging.

Both telescope backends are equipped with quadrature hybrids which can be used to generate circular polarisations. Unfortunately, when this is used the calibration is only valid across a narrow frequency range ($\sim 30$ MHz) which makes accurate calibration across the GNSS frequency range impossible. To avoid introducing frequency-dependent and potentially time variable elliptical polarisation into the correlation, we have instead bypassed the quadrature hybrid and recorded only the orthogonal linear polarisations. This does introduce a delay difference in the signal paths of the X and Y polarisations at both Hobart26 and Ceduna.

Neither telescope has well-defined polarisation characteristics, and both have unusual optics. While Hobart26 is an XY-mount telescope with a prime focus receiver, Ceduna’s L-band system is implemented with a tertiary reflector and receiver mounted on the dish surface. Neither receiver has been calibrated for polarisation leakage.

**Fig. 9:**
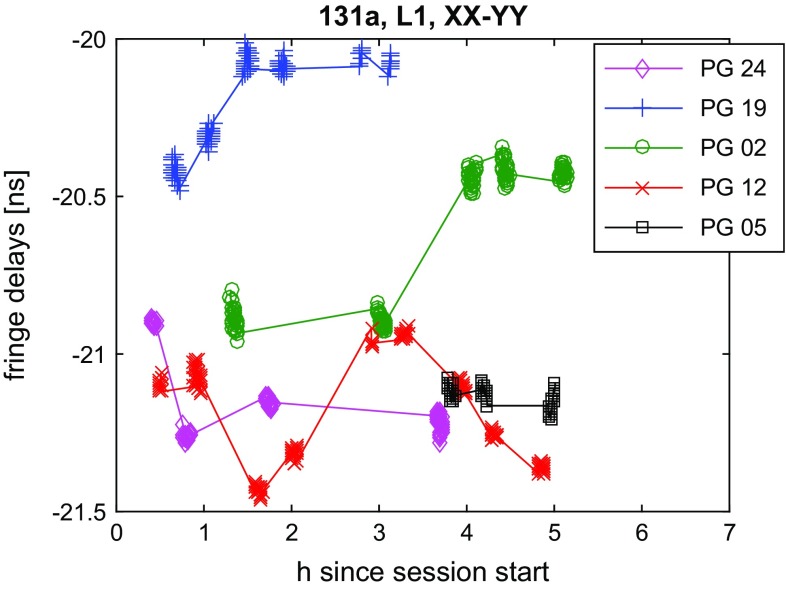
Fringe delays in L1 of 131a. Difference between the XX- and YY-polarised signal. Besides a large constant offset, variations specific to each of the observed satellites are visible

**Fig. 10:**
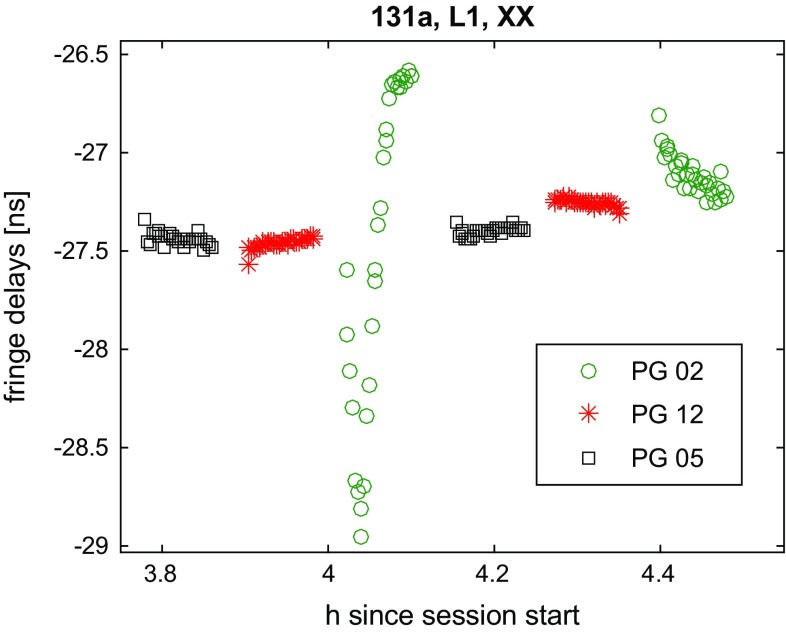
Fringe delays in L1 of 131a. An example for rapid variation in the XX fringe delays for one satellite track (PG02) is shown

Inspecting the data, we see that all polarisation products (XX, YY, XY, YX) have an approximately equal amplitude when observing the GNSS sources, due to the circularly polarised signal. By contrast, the quasars show clear signs of the relative orientation of the probes with the amplitudes swapping between the parallel and cross-hands during the observations. In comparing the GNSS data at the two recorded parallel-hand products, we find a small offset (of a few ns) between the polarisations (Fig. 9). These offsets are quite stable for the individual scans and should correspond to the different path lengths in the receiver. On closer inspection variations at the level of 1 to 2 ns between the XX- and YY-polarised signal are found, which are specific to each satellite. This indicates that the unsolved polarisation issue causes variations in the delays with the changing geometry of the baseline satellite constellation. Another effect is rapid changes in the measured delays for single satellite tracks, as illustrated in Fig. 10. These are also thought to be due to the linearly polarised signal or gain variations in one polarisation, as these artefacts often vanish in the other results using a different polarisation product.

Full polarisation calibration has not been possible with our current set of observations, as we have not optimised for parallactic angle coverage of the observed quasars. Whether the polarisation calibration obtained using quasar measurements would be valid when applied to the GNSS observations is also doubtful, given the large changes in the gain.

As we have equivalent amplitudes in each of the polarisation products for the GNSS sources and the following results are similar for the YY data, we have opted to focus on the XX product only in the following.

### Postprocessing

In the following analysis, only the satellite observations were used. While the observations of quasars were helpful for establishing a clock model and as a check of the system, their single-band delay precision is generally poor. Additionally, while in 126b and 131a the quasars were detected in the two satellite bands, no detections were found in one of the intermediate frequencies. The reason for this is unclear but may be due to a configuration error in the recording backend. As a consequence, the initial idea of estimating a multi-band delay from the quasar scans has not been implemented.

The satellite signal was detected in the allocated L1- and L2-bands. The SNR reported by fourfit is extremely high. In the L1-band, the SNR tended to be stable and consistent at $\sim 15000$ while the L2-band SNRs were somewhat weaker and considerable more variable ($\sim 4000$ to 10000). For comparison, the quasar scans show SNRs of 20 to 200 in L1 and 5 to 60 in L2.

As a result, the delay precision within a 5-min track in L1 is a bit better than in L2. Besides the higher noise, there are also significantly more severe problems for the individual tracks found for the L2 data, as jumps within a track of several ns. It shall be kept in mind that the L2 frequency is slightly out of the nominal range of the receivers. There are bandpass filters in the RF (radio frequency) chain which may be causing nonlinear phase responses for the L2 signal. This needs further investigation. As result, a combination of the L1 and L2 data is not found to be feasible yet.

For similar reasons, the GLONASS data are not discussed further here. Despite fringe delay detections in both bands, the results are more noisy and show more instabilities.

For the further analysis only GPS data are used, namely the XX polarisation product observed in the L1-band.

## Analysis

In this section the analysis chain for the VLBI satellite observations is introduced. While the data are not sufficient yet for presenting geodetically meaningful results such as station positions or orbit parameters, the necessary tools have been developed and can be tested with these data.

### Analysis software

We use the VieVS2tie component of the Vienna VLBI software VieVS. Detailed documentation including the mathematical formulations can be found in Plank (2014) and Plank et al. (2014). The main features are a new input format (VSO) for theses satellite observations, the handling of orbiting satellites as targets (via SP3-files) and a near-field delay model based on a light time solution of travel times. In principle VieVS2tie can handle both satellite and quasar targets in the same session including the partial derivatives for the estimation of the model parameters. Here we only use satellite observations due to the low quality of the quasar observations.

### Residual delays

**Fig. 11:**
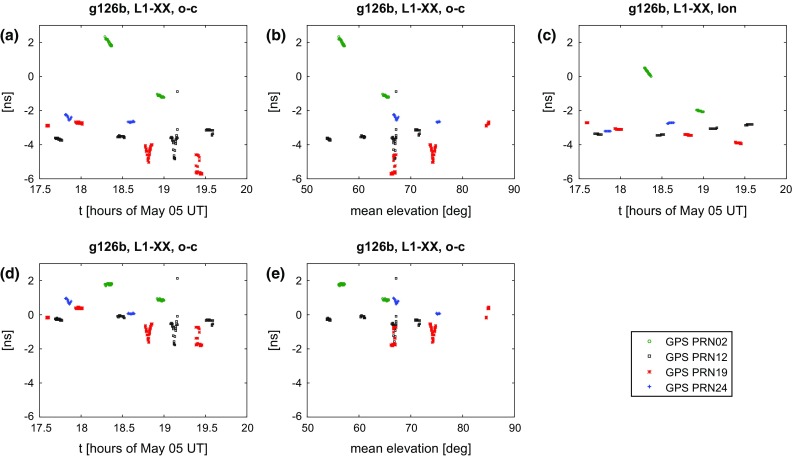
Residual delays (observed minus computed, o−c) for 126b. A clock offset and rate was estimated and removed. In the top line we show the residual delays versus time **a** and elevation **b**, as well as an estimate of the contribution due to the ionosphere calculated using TEC maps **c**. In the bottom line the residuals after applying the ionospheric model are shown **d**, **e**

**Fig. 12:**
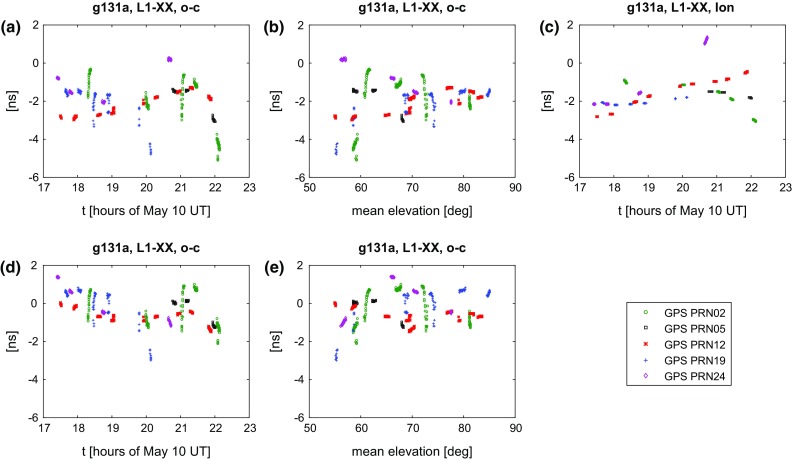
Same as Fig. 11, for 131a

From the correlation, together with the a priori model, we have total observed delays. Time epochs are the times of signal reception at station one, calculated at integer seconds. An initial check of the data quality can be done by comparing the observed delay with the modelled one (observed minus computed, o−c). This is done in Figs. 11 and 12. As we used the same software for creating the a priori delays as for the analysis, the residual delays are in principle the fringe delays we obtained during correlation. One exception is the clock model, with a clock offset and rate removed in the following analysis.

For 126b and 131a we find residuals within $\sim$8 ns or $\sim$2.5 m for the observed four or five satellites over the entire session of 2.5–6 h. Hereby the residuals of scans to the same satellite show an orbital signal, indicating shortcomings in the modelling. Generally, the residuals within a 5-min scan are highly correlated and are much smaller. As already mentioned in Sect. 5, some scans show considerable variation or rapid change in the residuals, which we believe is a result of the unresolved issues with gain and polarisation. The reader shall be reminded that, due to instrumental delays and clock offsets, our observations do not include information about the absolute residuals and the shift on the y-axis is arbitrary. It is encouraging to note that the estimated clock rate is identical for both sessions, 126b and 131a, which are five days apart.

Plotting the residuals versus the mean elevation $(el_1+el_2)/2$ reveals larger (absolute) residuals at lower elevations. This is relatively clear in Fig. 12b, while in 11b this mainly applies to a single satellite, PRN02. We think this elevation dependency is strongly indicating that it was caused by propagation medium including atmosphere and ionosphere. While the hydrostatic contribution of the troposphere is modelled, there is no correction of the wet troposphere nor for the ionosphere included in the model yet. VieVS has the possibility to calculate the delay due to the ionosphere using global maps of the total electron content (TEC) in the atmosphere (Tierno Ros et al. 2011). The maps are provided by the IGS, based on observations of the GNSS. Using the GPS L1 frequency, for 126b and 131a which were observed at local nighttime, ionospheric delays of up to 4 ns are found.

Applying this correction, the overall residuals drop to within $\sim$4 ns or $\sim$1.2 m. After the ionospheric correction, most of the elevation dependency is gone and most of the scans that were initially highly variable now appear well behaved. This is illustrated in Figs. 11 and 12. In 131a, these are for example the scan to PRN24 around 20:30 UT or the final two scans around 22:00 UT to PRN02 and PRN05. Despite this obvious improvement, it also becomes clear that correcting for the effect of the ionosphere using global TEC maps does not necessarily cover all effects. Looking at PRN02 in 126b one sees that the large residuals get diminished but do not fully vanish after the correction. Also, while the residuals in 131a at high elevations initially line up quite nicely, applying the ionospheric correction seems to introduce an offset for PRN19. Further improvements might be achieved by applying more sophisticated ionospheric corrections (e.g. developed by Männel and Rothacher 2016, for exactly the purpose of VLBI satellite observations) or through ray tracing methods to account for the wet part of the troposphere (e.g. Nafisi et al. 2012).

These residual delays can then be used in a least-squares adjustment on geodetic parameters, which is also fully implemented in VieVS. However, due to the fact that we have single-baseline observations only and because the collected data still include some unresolved systematic effects, such estimates are not very meaningful and not further discussed here.

### Discussion

The analysis above shows that, despite some problems in observing and estimating the full transmitted signal after correlation, the achieved results are meaningful. While applying the ionospheric correction significantly improves the residuals, it seems that there are more un-modelled signals left in the observations. In the following a ”health check” of the geometrical model is performed with the goal to identify areas of inaccuracies and improvements for the future.

On the top of the list is certainly the application of phase centre offsets for the observed satellites. While the orbit position refers to the centre of mass, the transmitting antenna is usually not exactly at this point. Such offsets can be as large as $\sim$3 m (Schmid et al. 2016) which would cause a periodic signal in the delays. This is ignored in the current processing and might explain some of the residuals. Using orbit files referring to the antenna phase centre offsets rather than by default to the satellite centre of mass, the magnitude of this effect for the sessions 126b and 131a was found to be between 0.02 and 0.1 ns, or 3 cm maximum.

The satellite positions are taken from the SP3-files provided by the IGS, interpolated applying the commonly used 9 th-order lagrange method. Their formal accuracy (as stated by the IGS) is at the level of 2.5, respectively, 3 cm for GPS/GLONASS. It is also worth noting that we did not find a large effect between using the rapid orbit product or the final orbits, or using the products of a different analysis centre. Various orbit products were tried for 179a, revealing differences in the computed delay of about 2 ps for GPS satellites and 15 ps for GLONASS. This means, that for timeliness, the rapid orbits can be used for correlation while the more precise analysis can be repeated using the more precise final orbits later on.

The same conclusion holds for Earth orientation parameters: tests when using predictions rather than measurements revealed effects at the level of a few ps only.

Work has also been done on the delay model itself, in adding gravitational delays and refining the light time iteration and the calculation of the satellite position at the time of signal emission. However, the effects of all previous refinements on the calculated delay were under 10 ps, which is the estimate for the accuracy of our near-field delay model that we are confident to give at the moment.

Finally some words about the Ceduna telescope, which is not usually used in geodetic experiments: Absolute coordinates for Ceduna were determined by Petrov et al. (2009) and subsequently included in a global frame. While Ceduna was added to the Vienna Mapping Functions (VMF; Boehm et al. 2009) and the tidal ocean loading, there are no corrections for atmosphere loading or for thermal antenna deformation applied for Ceduna. Together with a nominal axis offset of 2  mm, which is also ignored in the current modelling, we estimate the coordinates of Ceduna to be precise at the level of a few centimetres.

## Outlook

The work described realises VLBI tracking of GNSS-satellites, from scheduling to analysis. While there are still many refinements necessary in all covered areas, the developed work flow represents a basis for more and improved observations.

As described above, the next immediate steps are a better calibration of the signal chain as well as a proper recording of the full circular polarised signal. Possibly other antennas are better equipped for this task. Future observations will be used for refinements in the tracking (continuous instead of stepwise) as well as for improved recording using an 8-bit mode and fixing the AGC in the DBBC. Our findings suggest that bandpass calibration, measured from quasar observations can be performed and may prove beneficial. Successful detection of the quasar signal in all bands will allow for a multi-band delay solution, making it possible to include the quasar observations in the final geodetic analysis.

While all experiments described above were made on a single baseline, adding a third antenna is a logical step. Besides the geometrical aspects allowing for a more meaningful geodetic analysis, a closed triangle will certainly help to distinguish between local instrumental and geometric baseline delays.

Finally, the developed techniques can also be applied to observations of new targets: besides other representatives of the GNSS, this can be the APOD satellite carrying a dedicated VLBI transmitter or even one of several geodetic space-tie satellites which are currently under discussion. For these, more research on the best characteristics of a dedicated VLBI signal will be necessary. Further, besides the classical VLBI constellation, observations using phase referencing or single dish code, respectively, Doppler measurements are worth to be investigated for geodetic purposes. If one is interested in determining the satellite position with VLBI, a proper orbit estimation tool will have to be developed.

## Footnotes

1
http://lupus.gsfc.nasa.gov/fsdoc/fshome.html.

2
http://www.haystack.mit.edu/tech/vlbi/hops.html.
